# Ultrasound-Sensitive Liposomes for Triggered Macromolecular Drug Delivery: Formulation and *In Vitro* Characterization

**DOI:** 10.3389/fphar.2019.01463

**Published:** 2019-12-05

**Authors:** Maria B. C. de Matos, Roel Deckers, Benjamin van Elburg, Guillaume Lajoinie, Bárbara S. de Miranda, Michel Versluis, Raymond Schiffelers, Robbert J. Kok

**Affiliations:** ^1^Department of Pharmaceutics, Utrecht Institute for Pharmaceutical Sciences (UIPS), Utrecht University, Utrecht, Netherlands; ^2^Imaging Division, University Medical Center Utrecht, Utrecht, Netherlands; ^3^Physics of Fluids Group, MESA+ Institute for Nanotechnology and Technical Medical (TechMed) Center, University of Twente, Enschede, Netherlands; ^4^R&D, ABNOBA GmbH, Pforzheim, Germany; ^5^Laboratory Clinical Chemistry & Hematology, University Medical Center Utrecht, Utrecht, Netherlands

**Keywords:** ultrasound-sensitive liposomes, high-intensity focused ultrasound, triggered drug release, macromolecule encapsulation and release, live-cell imaging, perfluorpentane

## Abstract

Mistletoe lectin-1 (ML1) is a nature-derived macromolecular cytotoxin that potently induces apoptosis in target cells. Non-specific cytotoxicity to normal cells is one of the major risks in its clinical application, and we therefore propose to encapsulate ML1 in a nanocarrier that can specifically release its cargo intratumorally, thus improving the efficacy to toxicity ratio of the cytotoxin. We investigated the encapsulation of ML1 in ultrasound-sensitive liposomes (USL) and studied its release by high-intensity focused ultrasound (HAccessedIFU). USL were prepared by entrapment of perfluorocarbon nanodroplets in pegylated liposomes. The liposomes were prepared with different DPPC/cholesterol/DSPE-PEG2000 lipid molar ratios (60/20/20 for USL20; 60/30/10 for USL10; 65/30/5 for USL5) before combination with perfluorocarbon (PFC) nanoemulsions (composed of DPPC and perfluoropentane). When triggered with HIFU (peak negative pressure, 2–24 MPa; frequency, 1.3 MHz), PFC nanodroplets can undergo phase transition from liquid to gas thus rupturing the lipid bilayer of usl. Small unilamellar liposomes were obtained with appropriate polydispersity and stability. ML1 and the model protein horseradish peroxidase (HRP) were co-encapsulated with the PFC nanodroplets in USL, with 3% and 7% encapsulation efficiency for USL20 and USL10/USL5, respectively. Acoustic characterization experiments indicated that release is induced by cavitation. HIFU-triggered release of HRP from USL was investigated for optimization of liposomal composition and resulted in 80% triggered release for USL with USL10 (60/30/10) lipid composition. ML1 release from the final USL10 composition was also 80%. Given its high stability, suitable release, and ultrasound sensitivity, USL10 encapsulating ML1 was further used to study released ML1 bioactivity against murine CT26 colon carcinoma cells. Confocal live-cell imaging demonstrated its functional activity regarding the interaction with the target cells. We furthermore demonstrated the cytotoxicity of the released ML1 (I.E., After USL were treated with HIFU). The potent cytotoxicity (IC_50_ 400 ng/ml; free ML1 IC_50_ 345 ng/ml) was compared to non-triggered USL loaded with ML1. Our study shows that USL in combination with HIFU hold promise as trigger-sensitive nanomedicines for local delivery of macromolecular cytotoxins.

## Introduction

Cytotoxins, like diphtheria, shiga, ricin, and mistletoe lectin-1 (ML1), are good examples of nature-derived macromolecules that display outstanding toxicity and, therefore, great potential for cancer treatment. They come from different natural sources but present a common bifunctional A–B structure and belong to the same class of ribosome inactivating proteins (type 2, RIP-II) (Cummings and Schnaar, 2017). Although these proteins vary in the specific mode of action, their cytotoxic effect on target cells follows three common steps: 1) B-chain mediated cell internalization, 2) translocation of the A-chain into the cytosol and 3) irreversible inhibition of the protein synthesis by the toxic polypeptide (Roberts and Lord, 1992). In particular, ML1 is the major active component of mistletoe extracts which are being used in adjuvant cancer treatment (Fritz et al., 2004; Horneber et al., 2008; Kim et al., 2012; Marvibaigi et al., 2014). The intravenous administration of crude extracts or purified lectins is not suitable owing to the severe risks created by their non-specific cytotoxicity for normal cells. Thus, the ability to exploit the potential of ML1 entirely depends on finding nanocarriers that can direct and localize its anti-cancer activity to tumors, while preserving healthy tissues.

Nanomedicine-based targeting approaches can increase the therapeutic index of drugs in two ways. First, they improve treatment localization and increase efficacy, while reducing toxicity to normal tissues. Second, the encapsulated drug compounds are protected from the degradation or elimination processes that naturally occur in a physiological environment. A good example of nanomedicines are liposomes, which can encapsulate both hydrophilic and hydrophobic drugs and be prepared using well-established techniques such as lipid-film hydration or remote loading (Allen and Cullis, 2013). The main drawback of these long circulating stealth formulations, like Doxil^®^, is the inadequate release of the drug within the tumor microenvironment: although there is high tumor accumulation of encapsulated drug, levels of free drug are only moderate, which limits the therapeutic efficacy. Thus, it is important to focus novel nanocarrier formulations that enable an active release mechanism rather than passive, spontaneous release of the loaded drug. If adequate release can be achieved intratumorally, the therapeutic availability can be restored once the nanomedicine has reached its intended target tissue (van Elk et al., 2016; Hua et al., 2018). Triggerable nanocarriers make use of endogenous or exogenous stimuli to release their cargo. Endogenous stimuli-responsive nanocarriers exploit factors associated with the tumor microenvironment such as low pH, redox gradients or the presence of specific enzymes. Exogenous-responsive nanocarriers respond to external stimuli such as temperature, light or ultrasound (Stylianopoulos and Jain, 2015; Wicki et al., 2015; Al-Ahmady and Kostarelos, 2016). In addition to small molecule delivery, recent reports have shown temperature-triggered drug delivery systems of macromolecules, including ML1 (Yuyama et al., 2000; Saxena et al., 2015; Huang et al., 2017; Matos et al., 2018). Such temperature-sensitive liposomes, however, showed only partial release of the macromolecular cargo: ca. 40% release of FITC-dextran 4 kDa and 10% release of ML1 (Matos et al., 2018). The current thermosensitive release hypothesis postulates that lysolipids form nanopores in the bilayer during the phase transition (∼42°C) through which the entrapped drugs can be released (Ta and Porter, 2013) in a size-dependent manner (Matos et al., 2018).

In view of the low release efficiency of temperature-sensitive liposomes, we aimed to develop an alternative nanocarrier system that releases macromolecular payloads more efficiently. We combine liposomes with perfluorocarbon (PFC) nanoemulsions thus creating ultrasound-sensitive liposomes (USL). Upon ultrasound-mediated activation the liposome-encapsulated PFC nanodroplet will vaporize and expand to produce a gas bubble, which will disrupt the liposomal bilayer and trigger drug release, as demonstrated in previous reports using small molecular weight drugs and aiming for intracellular delivery (Javadi et al., 2012; Pitt et al., 2014; Husseini et al., 2015). In the current manuscript, we first optimized the protocol for creating USL using the HRP as macromolecular payload model. Next, USL formulations of ML1 were prepared and evaluated for their encapsulation efficiency, acoustic response, and release by high-intensity focused ultrasound (HIFU). Lastly, we investigated whether ML1 released from ML1-USL is functionally active, by demonstrating its uptake in cancer cells and cytotoxic activity after HIFU triggered release.

## Materials and Methods

### Chemicals

The phospholipids 1,2-dipalmitoyl-sn-glycero-3-phosphocholine (DPPC), and 1,2-distearoyl-sn-glycero-3-phosphoethanolamine-N-PEG_2000_ (DSPE-PEG_2000_) were purchased from Lipoid (Ludwigshafen, Germany). Cholesterol, 3,3’,5,5’-Tetramethylbenzinide (1-Step™ Ultra TMB-ELISA Substrate Solution) and HRP were purchased from Sigma-Aldrich. Perfluoropentane, tech. 90%, was purchased from Alpha Aesar (Germany). ML1 reference standard for ELISA (4.5 µg/ml) was provided by ABNOBA GmbH (Germany). Anti-ML1 monoclonal antibodies with specificity to ML1 A-chain anti-ML-A-5F5, and anti-ML-A-5H8-HRP (POD) were obtained from SIFIN (Berlin, Germany). CellTiter 96^®^ AQueous One Solution Cell Proliferation Assay (MTS) was provided by Promega. The lipophilic fluorescent dyes 3,3′-dioctadecyloxacarbocyanine perchlorate (DiOC_18_(3); DIO’) and Alexa Fluor^®^ 647 were purchased from Invitrogen.

### Methods

#### Mistletoe Lectin-1 Isolation and Characterization

ML1 was isolated from mistletoe plant material as described before (Matos et al., 2018). In brief, ML1 was isolated by affinity chromatography from mistletoe plant material that was harvested in June from ash tree (*Fraxinus excelsior* L.). After purification, ML1 was characterized by FPLC using a Mono S cation exchange column (Pharmacia/GE Healthcare, Uppsala, Sweden) and a 0.6 M NaCl salt gradient in 0.015 M citrate buffer (pH 4.0) at a detection wavelength of 280 nm. For chromatograms, see (Beztsinna et al., 2018). ML1 concentrations were quantified by UV/Vis at 280 nm (NanoDrop ND-1000; Thermo Fisher Scientific) using an extinction coefficient of 104850 M^−1^cm^−1^. ML1 concentrations were also quantified by sandwich ELISA, as describe elsewhere (Eifler et al., 1993). Anti-ML-A-5F5 was used as trapping antibody while anti-ML-A-5H8-POD was used as detection antibody.

ML1 was fluorescently labeled with Alexa Fluor 647 (AF647) succinimidyl ester according to the manufacturer’s protocol. In brief, 250 µL of 0.02 M bicarbonate buffer pH 8.3 was added to 2 ml of ML1 5.6 mg/ml (12 mg, 0.2 µmol). The diluted protein was reacted with AF647 dye (1:5 protein/dye mol/mol ratio) under stirring at room temperature for 1 h and purified by dialysis (Slide-A-Lyzer 0.5 to 3 ml, MWCO 10000 Da). Purified AF647-ML1 was characterized by analytical size-exclusion chromatography on a Bio Sep 3000 column (20 min, PBS 1 ml/min) and NanoDrop (AlexaFluor extinction coefficient 239,000 M^−1^ cm^−1^). The typical final ML1:dye ratio was 2:1 (mol/mol). Labelled ML1 was kept protected from the light at 4°C until further use.

#### Preparation of Nanocarrier Formulations

**Figure 1:**
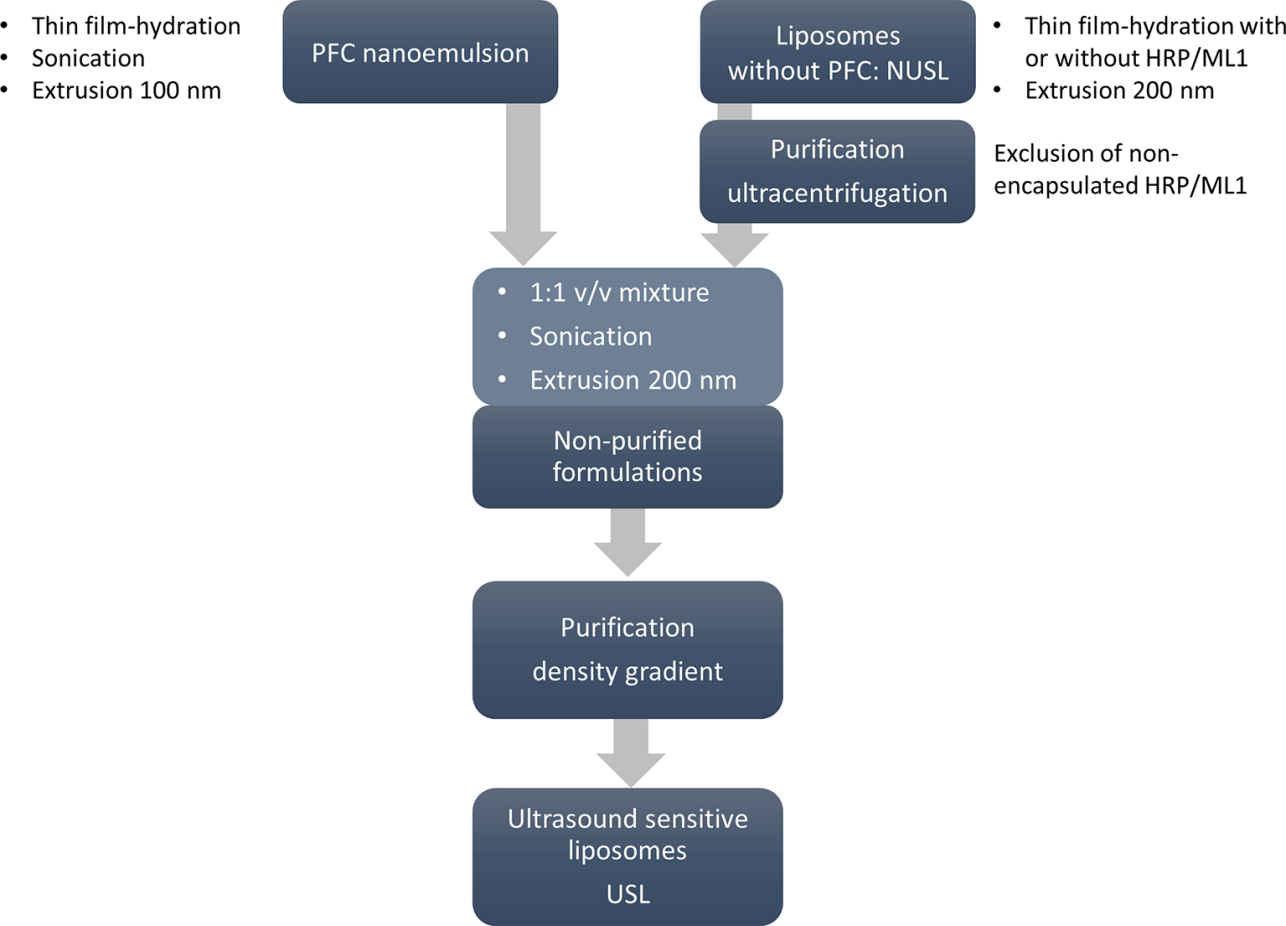
Workflow for the preparation of USL. First PFC/DPPC were prepared by thin film-hydration method and the resulting emulsion was downsized by sonication and extrusion through 100-nm pore size membrane. In parallel, the non-ultrasound sensitive liposomal formulations (NUSL; with and without cargo) were prepared by thin film-hydration and extrusion through 200-nm extrusion membranes and purified to remove the non-encapsulated cargo. Finally, PFC nanoemulsion and NUSL were mixed in the same volume ratio using sonication and one last step of extrusion through 200-nm pore size membrane. The formulations were purified by sucrose gradient to separate non-encapsulated drug, nanoemulsions, and empty liposomes from the final USL.

The preparation of USL involves several steps as depicted in the scheme below (**Figure 1**). Each of the steps is described in detail in the following sections.

##### Preparation of PFC Nanoemulsions

PFC nanoemulsions were prepared by thin film-hydration method (Javadi et al., 2012; Lattin et al., 2015). A lipid film containing 10 mg (15 µmol) of DPPC was prepared by evaporating the solvents from a 0.5-ml DPPC solution (20 mg/ml in chloroform) using a rotavapor at 60°C. The film was kept for 1 h in a nitrogen stream at room temperature before it was hydrated with 2 ml HBS (10 mM HEPES buffer pH 7.4 containing 150 mM NaCl) thus yielding DPPC vesicles with a final concentration of 7.5 mM; the resulting DPPC dispersion was cooled to 4°C on an ice bath. Perfluoropentane (0.6 ml; 3.5 mmol; density 1.6 g/ml) was added to the DPPC dispersion, and the mixture was sonicated (Branson Sonifier 20 kHz 13 mm) 3 times for 30 sec on the lowest energy input (10% duty cycle), with 1 min interval between each sonication. The resulting emulsion was extruded ten times over 100 nm polycarbonate extrusion membranes (Whatman) to reduce the size and narrow the polydispersity of the nanodroplets, as was confirmed by dynamic light scattering (DLS) using a Zetasizer Nano-S (Malvern Instruments).

##### Preparation of Liposomes

Liposomes with different lipid compositions were also prepared by the lipid film and extrusion method. DPPC/Cholesterol/DSPE-PEG_2000_ lipid molar ratios before combination with PFC nanoemulsions (named NUSL, non-ultrasound sensitive liposomes) were 60/20/20 for NUSL20; 60/30/10 for NUSL10; 65/30/5 for NUSL5; control liposomes were prepared with DPPC/Cholesterol/DSPE-PEG_2000_ 65/30/5 mol ratio only. For fluorescently labeled liposomes, 0.5 mol% of DIO’ was added when applicable. In brief, 80 µmol total lipid (TL) was dissolved in 4 ml 1:1 chloroform/methanol. Solvents were evaporated in a rotavapor for 20 min at 60 °C. The formed lipid films were kept for 1 h in a nitrogen stream and hydrated at 50°C with 1 ml HBS (in case of control liposomes) or HBS solutions of ML1 (1.5 mg/ml) or HRP (0.2 mg/ml). After reconstitution of liposomes, the final lipid concentration was 80 mM. Liposomes were extruded ten times over 400 and 200 nm pore-size polycarbonate filters at 45°C. Non-encapsulated ML1 and HRP were removed by ultracentrifugation of the liposomes (Beckmann ultracentrifuge, 2 cycles, 55000 rpm, 1 h, 4 °C) and resuspension in 1 ml HBS.

##### Combination of PFC Nanoemulsion and Liposomes into USL

USL were formed by mixing the DPPC-PCF5 nanoemulsion and the NUSL liposomes in a 1:1 volume ratio. The resulting solutions were sonicated (Branson Sonifier 20 kHz 13 mm) 3 times for 30 sec on the lowest energy input (10% duty cycle), with 1 min interval between each sonication, on ice bath. Finally, USL were extruded over 200 nm pore size polycarbonate membranes. The theoretical DPPC/Cholesterol/DSPE-PEG_2000_ lipid compositions of the final formulations (named USL, ultrasound sensitive liposomes) were USL20: 65/17/17; USL10: 63/30/7; USL5: 70/26/4. All USL formulations were purified by sucrose density gradient centrifugation to remove un-encapsulated drugs, nanoemulsions, and empty liposomes. In brief, sucrose solutions (10, 15, 20, 25, 40 and 50 w/w%) were prepared by dissolving pure sucrose in deionized water. The sucrose solutions with different mass fractions were carefully added to 15-ml ultracentrifuge tubes (Beckmann) in different volumes (2, 2, 2, 1, 1, 1 ml, respectively). Unpurified USL dispersion (1 ml) was carefully added to the top of the gradient and centrifuged at 35000 rpm for 16 h and 4°C (Beckmann ultracentrifuge). Non-encapsulated PFC nanoemulsion droplets have the highest density (1.6 g/ml) and settled at the bottom of the tube; free ML1 and emulsion-free NUSL had the lowest density and were collected in the upper sucrose layers. USL were recovered from the 20% sucrose layer. Isolated fractions were dialyzed against 2 L of HBS buffer for 24 h. USL and other fractions stored at 4°C until further studies.

### Characterization of PFC Nanoemulsions and Liposomes

#### Size and Polydispersity Index

The hydrodynamic diameter and polydispersity index of all lipid formulations were measured by dynamic light scattering using a Zetasizer Nano-S (Malvern Instruments). Appropriate dilutions were made in HBS buffer.

#### Lipid Recovery

The lipid recovery (TL) was determined by measuring the amount of phospholipids in 160 liposomal aliquots according to the method of Rouser et al. (1970). Sodium biphosphate was used as a reference. The blue colored reaction product was detected at 797 nm spectrophotometrically (SPECTROstar plate reader, BMG Labtech, Ortenberg, Germany).

#### Recovery of Payloads—HRP and ML1

An aliquot of 20 L of liposome dispersion was diluted in 1000 µL of HBS, to which TritonX-100 0.1% v/v was added to destroy the liposomal bilayer. HRP was determined enzymatically by eHRP–TMB reaction. In brief, 100 µL of HRP was added to the wells of a 96-well plate. The substrate (TMB, 25 µL/well) was added, and the mixture was allowed to react for 2 min 30 sec, after which the reaction was stopped by addition of 25 µL/well 1 M sulfuric acid. The yellow colored reaction product was detected at 450 nm with the spectrometric plate reader. ML1 was determined by sandwich ELISA as described above. Loading contents (LC%) and encapsulation efficiencies (EE%) were calculated as follows:

(1) $$LC\%=\;\frac{nmol\;payload}{nmol\;total\;lipid}\times 100$$

(2) $$EE\%=\;\frac{payload_{end}}{payload_{start}}\times 100$$

where, payload_end_ is payload of HRP or ML1 determined experimentally after formulation and purification, and payload_start_ is the starting amount of payload. Concentrations are expressed in µg/ml.

#### Transmission Electron Microscopy

The inclusion of the nanoemulsion in the liposomes was confirmed by negative staining transmission electron microscopy (TEM). In brief, NUSL or USL samples were placed on a carbon-coated copper grid (300 mesh; Plano GmbH, Wetzlar, Germany) and allowed to settle for 2 min before being blotted away by filter paper. An ammonium molybdate solution (1%) was added to the grid for 2 min, after which the solution was blotted away, and the grid was allowed to dry. Images were recorded at 120  kV on a Philips CM12 transmission electron microscope coupled to a GATAN Multiscan 400HP camera.

### Stability Studies and Release Experiments

#### Storage Stability

Storage stability of liposomes was assessed at 4°C and included colloidal stability (i.e., nanoparticle size and polydispersity) and drug retention capacity over a time period of 4 weeks. At each time point, small aliquots were diluted and analyzed by DLS or analyzed for released cargo (i.e., HRP) by enzymatic analysis. Drug retention capacity was calculated as follows:

(3) $$\text{Drug retention}\%=\frac{{\text{payload}}_{0}-{\text{leaked}}_{\text{t}}}{{\text{payload}}_{0}}\times 100$$

where *payload* *_(0)_* is the amount of encapsulated HRP at the initial timepoint of the stability study and leaked _(t)_ is the amount of HRP detected in the supernatant of the liposomes.

#### Ultrasound Triggered Release Experiments

Ultrasound-triggered release experiments were performed using an in-house developed HIFU setup (Oerlemans et al., 2013) consisting of a single-element US transducer (Imasonic, Besançon, France) placed inside a water bath containing a sample holder for a PCR tube. The PCR tube is positioned at the focal point of the single-element focused US transducer. The HIFU transducer had a focal length of 80 mm and a diameter of 120 mm. The pulsed (pulse repetition period = 50 ms, duty cycle = 1%) sinusoidal signal (1.3 MHz) was generated using a RF generator and amplifier (AG1021). The dimensions of the focal point were 1 × 1 × 3 mm (at −3 dB). Liposomal stock solutions (USL, NUSL), ML1 and HRP reference solutions and 1:1 v/v mixtures of PFC nanoemulsion plus NUSL were diluted 50-fold in HBS; 170 µl was transferred into the reaction vessel (170 µL PCR tubes; BioRad) and positioned in the HIFU setup. Samples were exposed to ultrasound (see exposure conditions below) and immediately thereafter transferred to an ice bath (4°C). Reference samples that had not been treated with ultrasound were kept at 4°C and were used as background levels of ML1 and HRP. In all cases, not more than 2% of background release was observed. Samples treated with TritonX-100 (0.1% v/v) were used as reference in which full release had occurred. Release of HRP was analyzed without further processing of the sampled aliquots. In the case of ML1, samples were processed by Vivaspin ultrafiltration (300 kDa MWCO; Sartorius) after which the ultrafiltrate was analyzed for released ML1 by ELISA as described above. In all release experiments, the percent release of the compounds was quantified by using the equation:

(4) $$\text{Release \%}=\;\frac{\text{amount released}}{\text{total release by TritonX}100}\times 100$$

where amount released is the amount of HRP or ML1 at a certain time point or fixed temperature, and total release by TritonX100 is the total mass found after liposomes were treated with Triton X-100.

##### HIFU Exposure Conditions

HIFU exposure conditions were optimized by (1) varying the peak negative pressure (2–24 MPa) conditions at constant exposure duration (1 min at room temperature), and (2) by varying the exposure time at constant peak negative pressures (5 or 24 MPa at room temperature). The samples were immediately transferred to the ice bath until further analysis as described above. Peak negative pressures in the focal point were calibrated as a function of input voltage using a fiber-optic hydrophone (Precision Acoustics) in a tank filled with water. The thermal effect of the HIFU exposure conditions were measured inside the PCR tube immediately after ultrasound exposure using a calibrated fiber optic thermometer (Neoptix, Canada).

#### Acoustic Characterization

##### Transducers

Three single-element transducers were used to characterize the acoustic behavior of nanoemulsions and liposomes: one for sonication, one for cavitation detection and one for attenuation measurement. The sonication transducer was calibrated using a fiber-optic hydrophone (Precision Acoustics). An arbitrary/function generator (WW1281A; Tabor Electronics) was utilized to generate twenty sinusoidal pulses of 100 cycles of 1.3 MHz at a pulse repetition frequency (PRF) of 100 Hz. This signal was then amplified using a 56-dB power amplifier (VBA100-200; Vectawave) and used to excite the transmitting C302 transducer (90% bandwidth, panametrics).

A passive receiving transducer (Vermon, SR 885C1001, Tours, France, 3 MHz, −6 dB relative bandwidth = 200%) was placed at 90° to the axis of the active sonicating transducer and detected acoustic emissions produced by the sonicated nanoemulsions and liposomes. Furthermore, a passive V304 (panametrics) receiving transducer was placed in line with active sonicating transducer to measure the attenuation of the transmitted signal due to scattering and/or absorption by nanoemulsions and liposomes. The signals collected from receiving transducers were acquired with a sampling frequency of 12.5 MS/s using a digital oscilloscope (TDS5034B; Tektronix, Beaverton, OR) and sent to a PC for analysis. Triggering was ensured by a pulse delay generator (Berkeley Nucleonics, model 575) controlled directly *via* a computer.

The experimental setup consisted of an acrylic tank filled with degassed water at room temperature, three transducers and an exposure chamber. The exposure chamber was aligned in the focal zone of the three transducers. The exposure chamber was designed using Solidworks, and printed with a 3D printer (RapidShape S30L). The front, back, and side of the sample chamber were covered with mylar with a thickness of less than 175 µm. The fourth side of the chamber contains a stirrer to avoid buoyancy affects or stagnation.

The exposure chamber was slowly filled with the nanoemulsions or liposome emulsions using a syringe. Subsequently, the sample was sonicated at 1.5 or 3.0 MPa with a twenty 100-cycle ultrasound pulses. After each measurement, the sample was removed from the exposure chamber, and the chamber was flushed with degassed, deionized water. All the experiments were performed at room temperature (20°C).

##### Cavitation and Attenuation Detection

**Figure 2:**
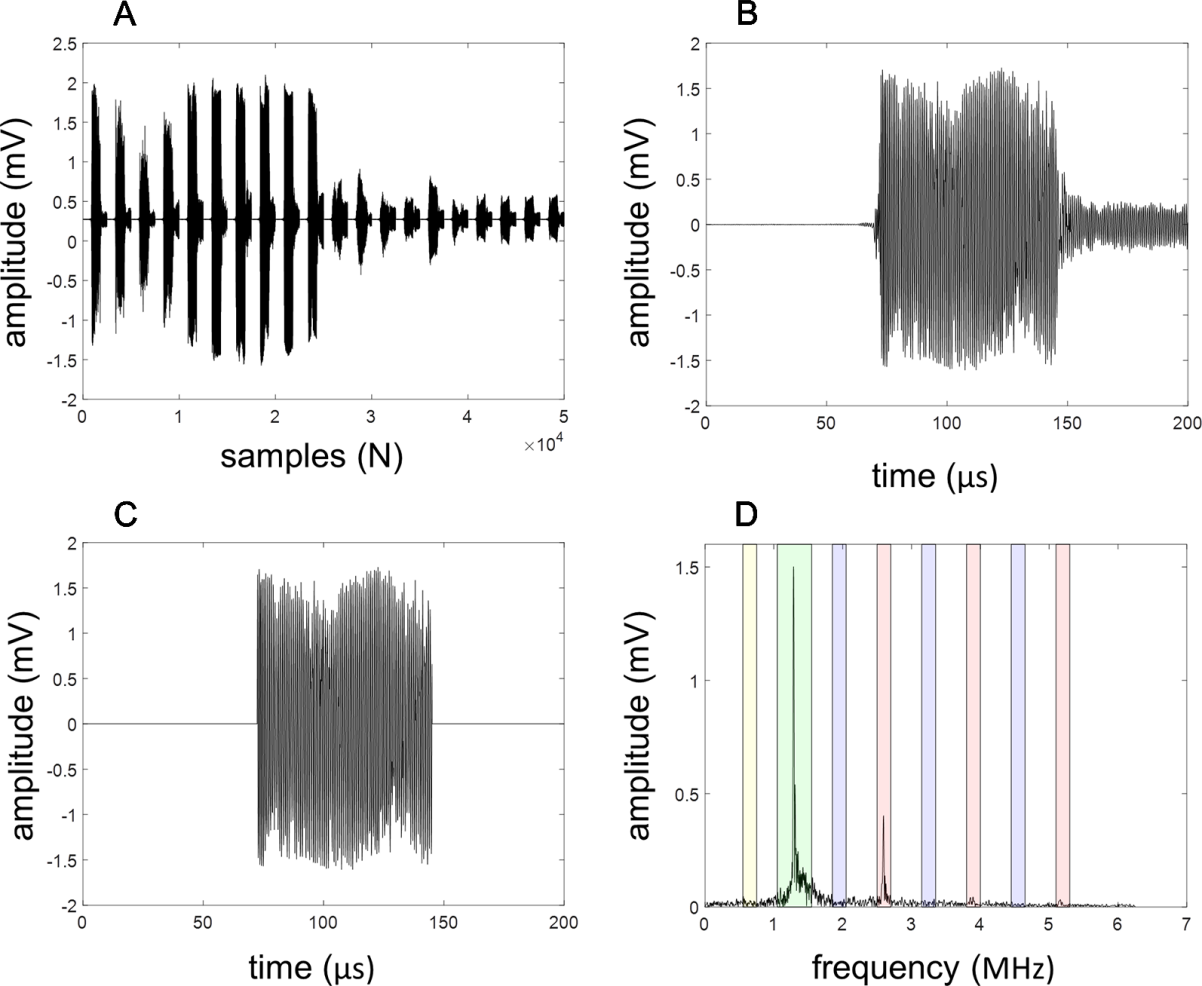
Illustration of acoustic characterization. A total of 20 pulses existing of 100 cycles were collected **(A)**. The detected signal from each pulse **(B)** is first trimmed **(C)** and subsequently converted to its frequency spectrum using a fast Fourier transformation **(D)**. The amplitude within the selected regions (red = harmonics, green subharmonic, blue = ultraharmonics) are calculated for each pulse and plotted as a function of number of pulses.

To analyze the acoustic emissions, the recorded data were processed using fast Fourier transform (FFT) analysis in MATLAB (MathWorks, Natlick, MA, USA) to create frequency spectra (see also **Figure 2**). The harmonics were defined as the maxima within ± 250 kHz around the harmonic frequency (n*f, f: excitation frequency, n = 1, 2, 3, or 4) in the frequency spectrum. The ultraharmonics were defined as the maxima within ± 100 kHz around the ultraharmonic frequency (m/2*f, f: excitation frequency, m = 3, 5, or 7) in the frequency spectrum. The subharmonics were defined as the maxima within ± 100 kHz around the subharmonic frequency (f/2, f: excitation frequency) in the frequency spectrum. Broadband noise was defined as the root-mean squared amplitude of the frequency spectrum after excluding the harmonics, ultraharmonics, and subharmonics as defined above. Attenuation, measured in dB, was calculated using the equation:

(5) $$L_{\text{dB}}=\;-20\cdot log10\frac{A}{{\text{A}}_{\text{ref}}}$$

with *A* the amplitude of the first harmonic of the transmitted signal with nanoemulsion, USL or NUSL in the exposure chamber and *A*_ref_ the amplitude of the first harmonic of the transmitted signal with water in the exposure chamber.

### Bioactivity of Free and Formulated ML1

Bioactivity of ML1 that had been encapsulated in liposomes and released by HIFU was studied in two experimental settings that reflect either the uptake of ML1 in target cells (functionality of the A-chain of ML1) or that represent the biological activity of ML1 (cytotoxin activity). All experiments were conducted with murine CT26 colon carcinoma cells that has been obtained from American Type Culture Collection (ATCC). RPMI cell culture media, PBS, and FBS were purchased from Sigma-Aldrich, and OptiMem was obtained from Gibco. CT26 cells were cultured in RPMI supplemented with 10% FBS, at 37°C in a 5% CO_2_ and humidiﬁed atmosphere. For all cell experiments, CT26 colon carcinoma cells were first seeded in 96-well plates (10000 cells/well) and allowed to adhere for 24 h prior to the experiment.

#### Uptake of Released ML1

Uptake studies were conducted with HIFU-treated DiO’-labeled liposomes (NUSL, USL, DiO’ at 0.5% mol of total lipid) that had been loaded with AF647-labeled ML1. Liposomes were diluted 1:10 in OptiMEM (final concentrations 2 g/ml AF647-ML1 and 7 mM total lipid) and treated with HIFU as described above with the following acoustic settings: 5 MPa for 1 min exposure and 24 MPa for 1 min exposure. Samples were transferred to the ice bath and used for uptake studies without further processing. Before adding the samples, nuclei of CT26 cells were pre-stained with Hoechst 33342. After replacement of the culture medium with the HIFU-treated samples, 96-well µClear^®^ black plates (Greiner) were transferred into a Yokogawa Cell Voyager CV7000s microscope (Tokyo, Japan). Live-cell confocal microscopy images were taken for 4 h at 37°C and analyzed for uptake of liposomes (red), uptake of ML1 (green), and nuclei (blue). Uptake was semi-quantified with Columbus^®^ image analysis software (PerkinElmer) using automated protocols for nuclei and cytoplasm detection and build-in functionalities for fluorescence intensity determination.

#### Cytotoxic Activity of ML1

Cytotoxic activity of ML1 was indirectly measured by a mitochondrial activity assay that quantified the number of surviving cells. For these experiments, non-labeled ML1 and non-labeled liposomes were used. ML1 containing liposomes (NUSL, USL, control liposomes) were diluted in RPMI+10%FBS (20 µl in 1000 µl) and 170 µl of the diluted sample was then exposed to HIFU as described above with the following acoustic setting: 5 MPa for 1 min exposure and 24 MPa for 1 min exposure. Samples were transferred to the ice bath and analyzed for cytotoxic activity after dilution of 50 µl of the samples with 80 µl of culture medium. The obtained samples were transferred onto the cells and incubated under culture conditions for 4 h.

Final concentrations incubated with the cells were 80 to 800 ng/ml for ML1 and 0.4 to 8 mM TL. After refreshing the media with drug-free culture medium, cells were cultured for an additional 44 h in the incubator before determining the number of surviving cells according to the supplier’s instruction (CellTiter 96^®^; AQueous One Solution Cell Proliferation Assay). Bioactivity IC_50_ values of each treatment were calculated by non-linear dose-response curve fitting using GraphPad Prism software. Appropriate reference samples included free ML1, blank liposomes, and samples not treated with HIFU ultrasound.

### Data Analysis

Data are presented as the average and standard deviation of three independent experiments with triplicate samples unless stated otherwise. Data were statistically tested in GraphPad Prism 7 (Graph-Pad Software, Inc, San Diego, CA) by comparison of groups with different tests (see figure captions for details of performed tests). Differences between groups with *p* < 0.05 were considered statistically significant.

## Results and Discussion

### Characterization of PFC Nanoemulsions and Liposomes

The final size of PFC nanoemulsion was 118 ± 11 nm (PDI, 0.26 ± 0.01) which is the expected size range after extrusion over 100 nm filters. Although PFC nanoemulsions were relatively stable. The size and PDI of PFC nanodroplets doubled upon storage at 4°C in 48 h, and after 4 days the size had increased drastically (1120 nm; PDI, 1.0). We therefore systematically used freshly prepared PFC nanoemulsions for the experiments.

The characteristics of NUSL and the corresponding USL are shown in **Table 1**. Before their loading with PFC nanodroplets NUSL10 and NUSL5 displayed sizes and PDI within the expected range (size 156–191 nm, PDI 0.11-0.09). NUSL20 showed sizes two times smaller than expected (i.e., 95 nm instead of ∼200 nm), which may be related to the formation of DSPE-PEG_2000_ micelles due to very high concentrations of this lipid in the formulation (Johnsson and Edwards, 2003; Johansson et al., 2005; Sandström et al., 2008; Evjen et al., 2010; Vainikka et al., 2011). Although one would expect an increase in PDI for such a mixture of two subsets of nanoparticles, the DLS-algorithm based single population analysis is unable to resolve this accurately (van Gaal et al., 2010). Considering the standard deviations, similar encapsulation efficiencies for HRP and ML1 were found for all NUSL formulations, i.e. no specific trend was observed towards the lipid composition.

**Table 1:** Physicochemical characteristics and loading results of NUSL and USL.

Formulation		Size, nm	PDI	TL, %	EE%	LC µg drug: µmol lipid
NUSL HRP	NUSL20	95 ± 1	0.12 ± 0.02	58 ± 1	29 ± 1	3.7 ± 0.2
	NUSL10	156 ± 2	0.11 ± 0.01	63 ± 2	40 ± 1	4.6 ± 0.3
	NUSL5	191 ± 4	0.09 ± 0.03	62 ± 2	25 ± 3	3.6 ± 0.1
USL HRP	USL 20	98 ± 2	0.17 ± 0.02	12 ± 1	3 ± 1	1.1 ± 0.3
	USL10	209 ± 13	0.15 ± 0.02	17 ± 1	7 ± 1	2.0 ± 0.1
	USL5	181 ± 1	0.06 ± 0.05	11 ± 1	7 ± 1	3.2 ± 0.2
NUSL ML1	NUSL20	97 ± 2	0.11 ± 0.03	66 ± 2	21 ± 1	4.1 ± 0.1
	NUSL10	186 ± 4	0.12 ± 0.01	72 ± 1	23 ± 1	4.5 ± 0.1
	NUSL5	179 ± 5	0.07 ± 0.04	59 ± 2	21 ± 1	4.7 ± 0.2
USL ML1	USL 20	88 ± 8	0.16 ± 0.01	18 ± 1	2 ± 1	2.9 ± 0.4
	USL10	179 ± 1	0.15 ± 0.01	14 ± 2	4 ± 1	6.7 ± 0.6
	USL5	201 ± 8	0.07 ± 0.03	15 ± 1	4 ± 1	6.5 ± 0.4

Lipid molar ratio of DPPC/Cholesterol/DSPE-PEG2000 for (N)USL20: 60/20/20; for (N)USL10: 60/30/10; for (N)USL5: 65/30/5. TL%, total lipid yield; EE%, encapsulation efficiency; LC, loading content expressed as µg drug to µmol lipid ratio. Average ± standard deviation of 2 independent samples.

After mixing the PFC nanoemulsion with the NUSL by sonication to enable the inclusion of PFC nanodroplets, the now-formed USL were extruded again to reassure a monodispersed size distribution. The average size remained unchanged but we observed an increase in PDI by ca. 2-fold (not shown), as anticipated by the fact that it is a mixture of two populations with different sizes.

**Figure 3:**
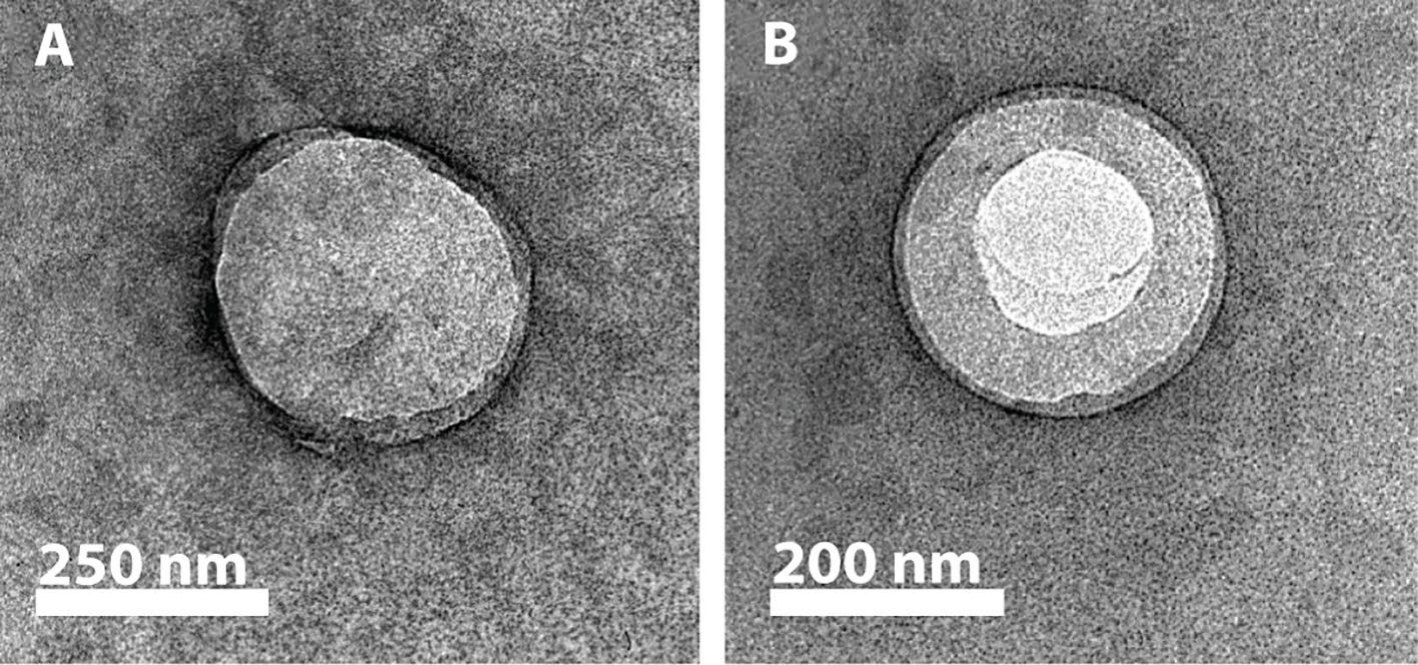
Representative TEM images of negatively stained NUSL10 **(A)** and USL10 **(B)** after purification. The images show the absence (NUSL) and the presence (USL) of nanoemulsion in the core of the resulting liposomes.

USL were separated from non-encapsulated nanoemulsion and non-encapsulated HRP or ML1 by sugar density gradients. The top layer contained mainly non-encapsulated proteins, fraction 1 (10% sucrose) contained purified NUSL, fraction 2 (20% sucrose) contained purified USL and the bottom fraction (50% sucrose) contained the nanoemulsion. Fraction 2 or USL was dialyzed to replace the external sugar solution by fresh HBS. The sizes and PDI of the final preparations were comparable to their corresponding NUSL formulation. When comparing the PDI of purified USL with the PDI of the USL before sucrose gradient purification, the decrease in PDI suggests that we have removed the non-encapsulated PFC nanodroplets and that we obtained a monodisperse sample. The encapsulation of the nanoemulsion was further confirmed by TEM (**Figure 3**). The recovery of lipids (TL%) and encapsulated cargo (EE%) decreased substantially when NUSL were converted into USL, but the LC remained constant. We attribute the numerical decrease to the USL formation process: to prepare USL, that is, to incorporate the PFC nanoemulsion in the liposomes, it is necessary to apply sonication to the NUSL already encapsulating the drug. This step includes not only transient opening of the NUSL bilayer and thus loss of some of the encapsulated cargo, but also partial replacement of the internal volume by PFC nanoemulsion. Moreover, the purification over sugar density gradients also removed liposomal vesicles that had not been loaded with PFC, which was responsible for ∼80% loss in recovery of both phospholipid and loaded drugs. Since LC% were not affected largely, it can be inferred that the loss of drugs was primarily related to low inclusion of PFC and removal of NUSL, rather than leakage of HRP or ML1 during sonication and extrusion.

### Stability Studies and Release Experiments

#### Storage Stability

**Figure 4:**
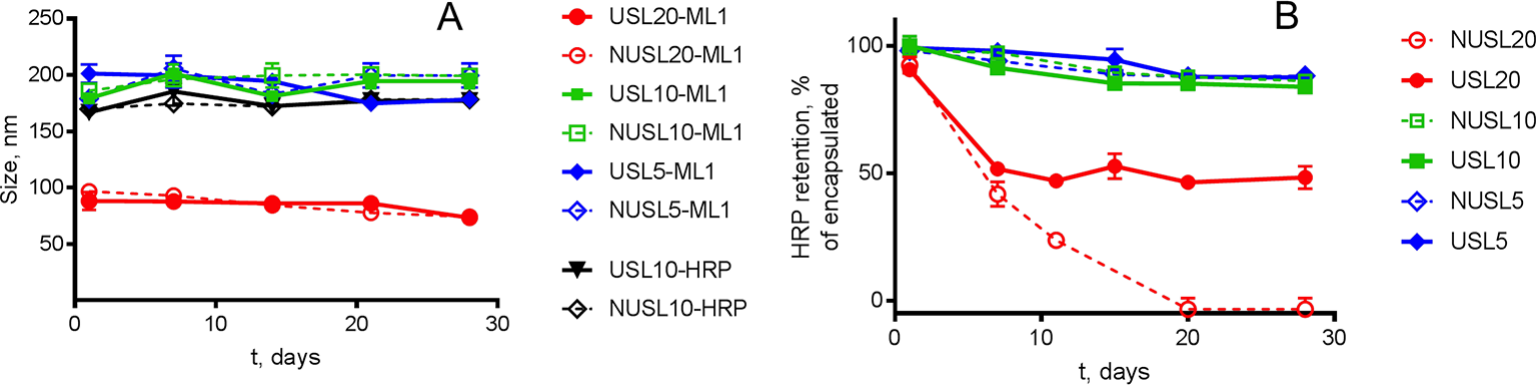
Storage stability of USL at 4°C in HBS buffer. Panel **(A)** shows size measurements, obtained by DLS over a period of 30 days, of nanoparticles (NUSL and USL) loaded with HRP and ML1. Panel **(B)** shows HRP retention. In both graphs, the colors of the lines and the symbols correspond to the same lipid composition: the red line corresponds to liposomal formulations composed of initial 20 mol% DSPE-PEG_2000_ and containing (full line and circle, USL20) or not (dashed line and empty circle, NUSL20) PFC nanoemulsion; the green line corresponds to liposomal formulations composed of initial 10 mol% DSPE-PEG_2000_ and containing (full line and square, USL10) or not (dashed line and empty square, NUSL10) PFC nanoemulsion; the blue line corresponds to liposomal formulations composed of initial 5 mol% DSPE-PEG_2000_ and containing (full line and diamond, USL5) or not (dashed line and empty diamond, NUSL5) PFC nanoemulsion. Data are the average ± standard deviation of three independent samples.

Formulations containing 5 and 10 mol% DSPE-PEG_2000_ with and without PFC nanoemulsion, i.e. (N)USL5 and (N)USL10, were stable with respect to particle size (**Figure 4A**) as well as drug retention (**Figure 4B**) when stored at 4°C for 1 month. On the contrary, (N)USL20 leaked 50% of the loaded HRP in the first week of storage, and also showed decreases in size revealing colloidal instability. This can be related to the amounts of 15% to 20% DSPE-PEG_2000_ in the (N)USL formulation. DSPE-PEG_2000_ amounts above 12 mol% are known for the formation of micelles and increased instability of liposomal bilayers (Johnsson and Edwards, 2003; Johansson et al., 2005; Sandström et al., 2008; Evjen et al., 2010; Vainikka et al., 2011). Since both NUSL20 and USL20 showed similar leakage in the first week of storage, PFC nanoemulsion does not seem to play a role in the instability.

#### Acoustic Characterization

**Figure 5:**
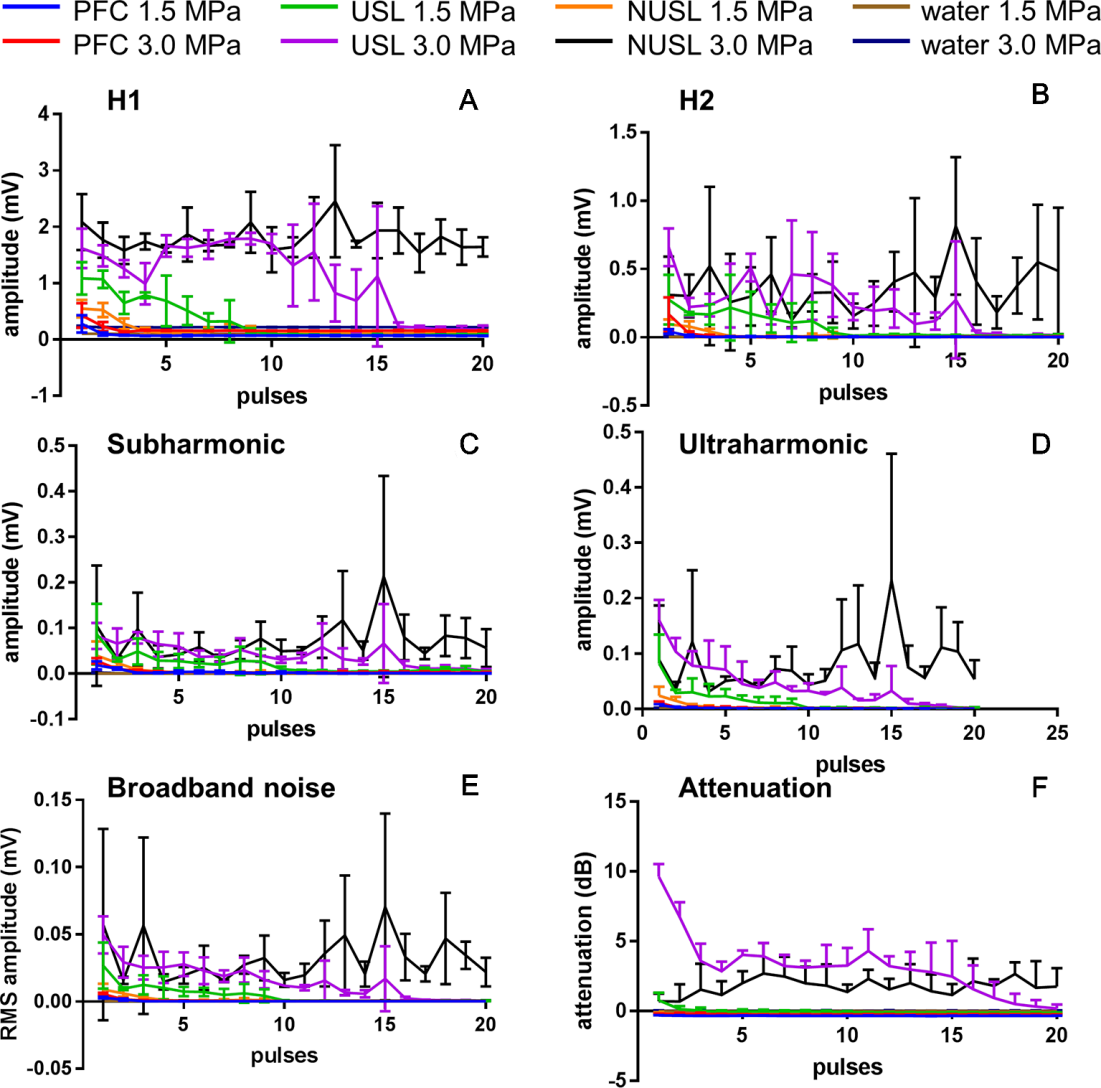
Acoustic characterization of PFC nanoemulsion, USL10 and NUSL10 at 2 acoustic pressures (1.5 and 3.0 MPa). The amplitude of the first **(A)** and second **(B)** harmonic frequency and of the subharmonic frequency **(C)** and ultraharmonic frequency **(D)**, the RMS value of the broadband noise **(E)** of the scattered signal as well as the attenuation **(F)** of the transmitted signal were measured. N = 3.

**Figures 5 A–D** depict the first harmonic (H1, 1.3 MHz), second harmonic (H2, 2.6 MHz), subharmonic (0.65 MHz) and broad band noise responses, respectively, from the PFC nanoemulsion, USL10, NUSL10, and water. The second harmonic, subharmonic, and broadband noise signals emitted by USL were clearly stronger as compared to signals emitted by PFC nanoemulsions, NUSL, and water. The presence of broadband emission is characteristic for inertial cavitation and therefore indicates that cavitating microbubbles were formed within the USL10 sample. The harmonic, subharmonic, and broadband noise signals of USL were pressure dependent and gradually decreased in time. As the peak negative pressure was increased from 1.5 MPa to 3.0 MPa the non-linear acoustic emission signals of USL increased in amplitude and remained elevated over a longer duration, i.e. more pulses. The gradual decrease of acoustic emission with increasing number of pulses sent is likely related to the depletion of the sample by disrupting particles that were activated by preceding pulses and activating new particles that are less responsive. The PFC nanoemulsions also showed some non-linear acoustic emission signals during the first pulses, whereas the non-linear acoustic emission signals of water were absent, as expected. Exposure of NUSL10 to elevated peak negative pressure (3.0 MPa) also causes inertial cavitation, but the activity did not decrease in time, by opposition to all other emulsions. **Figure 5E** shows the attenuation, measured in dB, of the transmitted signal for the PFC nanoemulsion, USL10, NUSL10, and water. For the USL the attenuation declined with increasing number of pulses, mirroring the acoustic scattering, i.e. the sample is depleted by the disruption of the particles. PFC and NUSL samples did not cause significant attenuation of the ultrasound pulses, except for NUSL10 at 3.0 MPa. The origin of this increased and sustained attenuation and scattering will deserve further investigation in the future. Beyond the acoustic characteristics, these measurements provide proof regarding the formation of cavitation bubbles from the emulsion. These microbubbles, in all likelihood, play a major role in the observed drug release.

#### HIFU-Triggered Release Experiments

**Figure 6:**
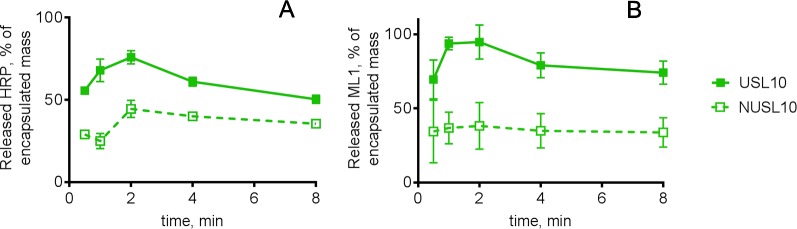
HRP and ML1 release from USL10 and NUSL10 as function of exposure time at fixed negative pressure (24 MPa), n = 4. In both graphs, the full line and full squares correspond to USL10, and the dashed line and empty squares correspond to NUSL10. For each exposure time the difference in HRP **(A)** or ML1 **(B)** release between USL10 and NUSL10 samples was significant (multiple t-test for all time points with Holm-Sidak correction).

**Figure 7:**
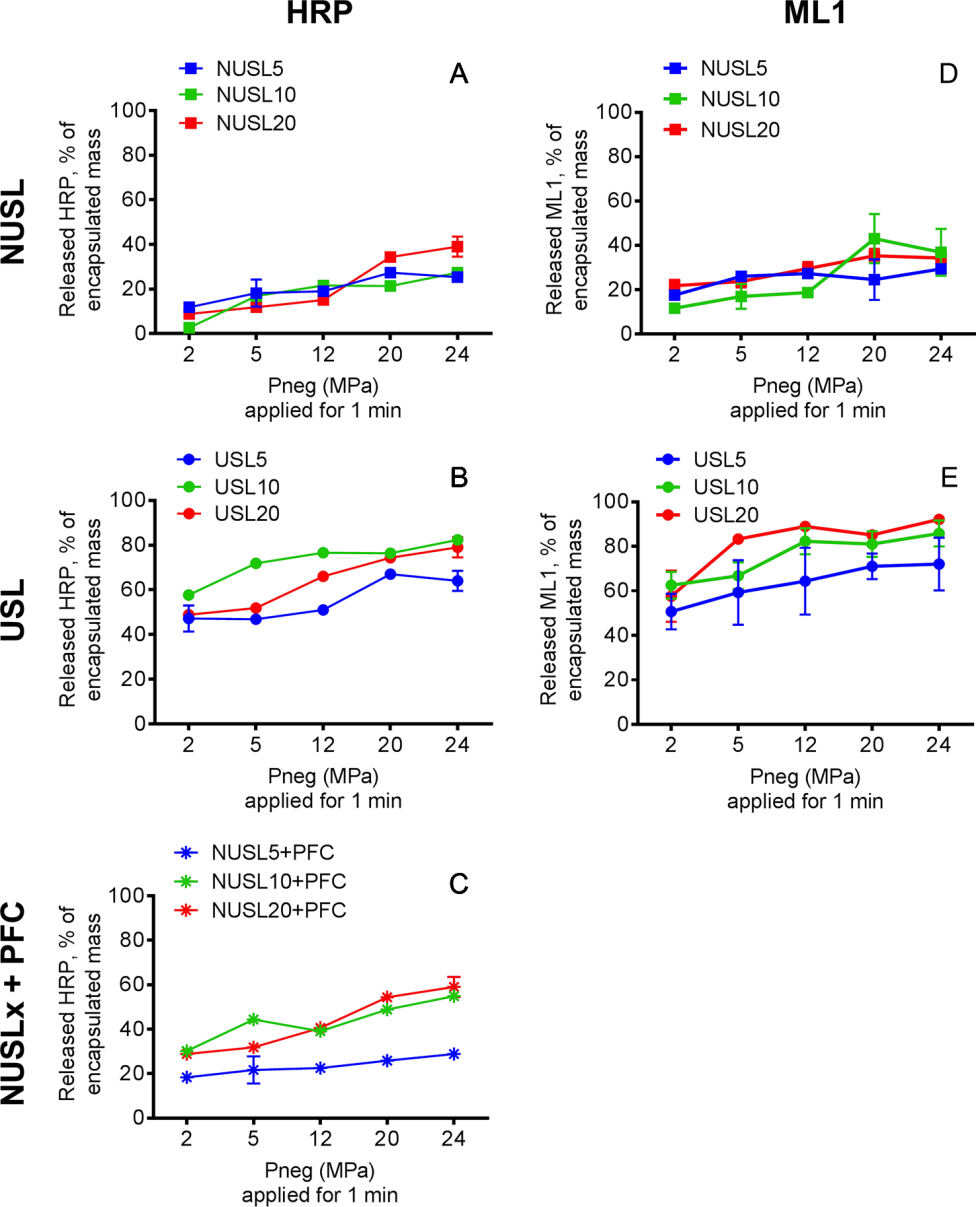
HRP release profiles from **(A)** NUSL (squares), **(B)** USL (circles) and **(C)** NUSL (stars) spiked with nanoemulsion, and ML1 release profiles from **(D)** NUSL formulations (squares) and **(E)** USL. All samples were exposure to HIFU for 1 min and variable negative pressure (2–24 MPa) and subsequently analyzed. In all graphs, the colors of the lines correspond to the same lipid composition: the red line corresponds to liposomal formulations composed of initial 20% mol DSPE-PEG2000; the green line corresponds to liposomal formulations composed of initial 10% mol DSPE-PEG2000; the blue line corresponds to liposomal formulations composed of initial 5% mol DSPE-PEG2000. Background release (i.e., without HIFU) was insignificant (<2%) for the tested conditions. Data are the average ± standard deviation of three independent samples. A 2-way ANOVA with Tukey’s multiple comparisons test showed that at each pressure and for each lipid composition the HRP release from USL was significantly higher compared to NUSL and NUSLx + PFC. Similarly, a 2-way ANOVA with Sidak’s multiple comparisons test showed that at each pressure and for each lipid composition the ML1 release from USL was significantly higher compared to NUSL.

To investigate HIFU-triggered release of macromolecular drugs from USL, we assessed acoustic time- and pressure-dependent release of HRP and ML1 (**Figures 6** and **7**, respectively). **Figure 6** shows the release of HRP (panel A) and ML1 (panel B) from USL10 over time at fixed peak negative pressure (24 MPa). With both macromolecules as cargo, we obtained highest release for 1 to 2 min HIFU exposure time. At higher acoustic pressures or longer exposure times, the percentage of HRP and of ML1 recovered decreased, and we speculate that it is due to the cargo damage (not shown).

Remarkably, NUSLs also showed HIFU triggered release, although the released amounts were considerably lower than observed for USL. It has been previously observed that normal liposomes can respond to ultrasound fields (Evjen et al., 2010; Oerlemans et al., 2013). Doxorubicin was released from such so-called sonosensitive liposomes (DSPE/DSPE-PEG_2000_/Chol 62:8:30 mol%) 7-fold more than from reference doxorubicin-loaded liposomes (HSPC/DSPE-PEG_2000_/Chol 57:5:38 mol%) (Evjen et al., 2010). For USL, however, bubble nucleation is promoted directly within the liposomes by the low stability perfluorocarbon, which explains a much more important release. As a result, larger macromolecules like HRP and ML1 can be released from DSPE-PEG_2000_-containing liposomes but the release extent can be significantly improved by incorporating the PFC nanoemulsion.

To investigate whether the HIFU-triggered release of USL really depends on the inclusion of PFC nanodroplets inside the liposomes, we evaluated the release of HRP from mixtures of NUSL and PFC nanoemulsions. Since we did not further treat the mixture of PFC and NUSL to promote encapsulation of the PFC nanodroplets, this formulation represented the physical mixture of two different nanoparticles, rather than a combined nanoparticle. HRP release after different peak negative pressures (2–24 MPa) and fixed exposure time (1 min) is shown in **Figures 7A–C**. The USL formulations showed superior release, when compared to the NUSL formulations or the NUSL mixed with nanoemulsion. The formulations containing emulsion only on the outside (NUSL*x*+PFC) showed an intermediate release performance. This confirms the importance of the presence of cavitation-promoting PCF directly inside the liposomes. Intermediate response resulting from the presence of the nanoemulsion near the liposomes is influencing drug release, possibly by enhanced energy transfer to the lipid bilayer that can deform or disrupt the bilayer. The phase transition of PFC from liquid to gas, which is in the range of 1 to 10 MPa (Aliabouzar et al., 2018), and the resulting expansion seems a plausible mechanism for the more efficient payload release. Thermal effects can be excluded since the maximum temperature increase upon HIFU exposure did not exceed 2 degrees (data not shown). USL20 and USL10 released the highest amounts of HRP while USL 5 released the least of the three formulations. However, USL20 showed the least storage stability, as was previously noticed. Overall, USL10 stands out as the best formulation and emphasizes the need to have the nanoemulsion inside the liposomes to maximize ultrasound-triggered release. The lower pressures (i.e. 2 and 5 MPa) cause already a significant macromolecular drug release (>50%) and can most likely be used *in vivo* without causing adverse events. In contrast, care has to be taken when the higher pressures are used *in vivo* since these pressures may cause undesired tissue damage (Health Protection Agency, 2010).

USL5 and USL20 containing ML1 were additionally tested for HIFU-triggered release at the lowest range of acoustic pressures and the results for ML1 release are summarized in **Figures 7D, E**. Similar to HRP-loaded USL formulations, USL5 released less ML1 than its counterparts USL20 and USL10 and we believe this can be attributed to the higher stability provided by a smaller amount of DSPE-PEG_2000_, as discussed previously. In conclusion, the formulations can be ordered according to their overall performance: USL20 < USL5< USL10. We chose USL10 carry on the *in vitro* bioactivity and continued our experiments using 1 min exposure time and 5 and 24 MPa peak negative pressures. Both pressures are significantly above the cavitation threshold, see section 3.2.2.

### Bioactivity of Formulated ML1

To investigate the overall bioactivity of the formulated ML1 we studied two phenomena related to the functionality of the protein. ML1 is composed of a cytotoxic A-chain linked to the lectin B-chain responsible for cellular binding and for mediating the protein uptake (Pizzo and Maro, 2016). It is therefore imperative to ensure that the structure of the protein is conserved to maintain its cytotoxic capacity. Taking this in mind, we studied both uptake of ML1 in CT26 cells and its cytotoxic activity. Uptake of ML1 was visualized by live-cell confocal fluorescence microscopy, using fluorescently labeled ML1 loaded in fluorescently labeled liposomes. CT26 cells were incubated for 4 h with HIFU-treated formulations, and the released ML1 induced cytotoxicity was measured 48 h later, as described before (Beztsinna et al., 2018; Matos et al., 2018).

#### Uptake of Released ML1

**Figure 8:**
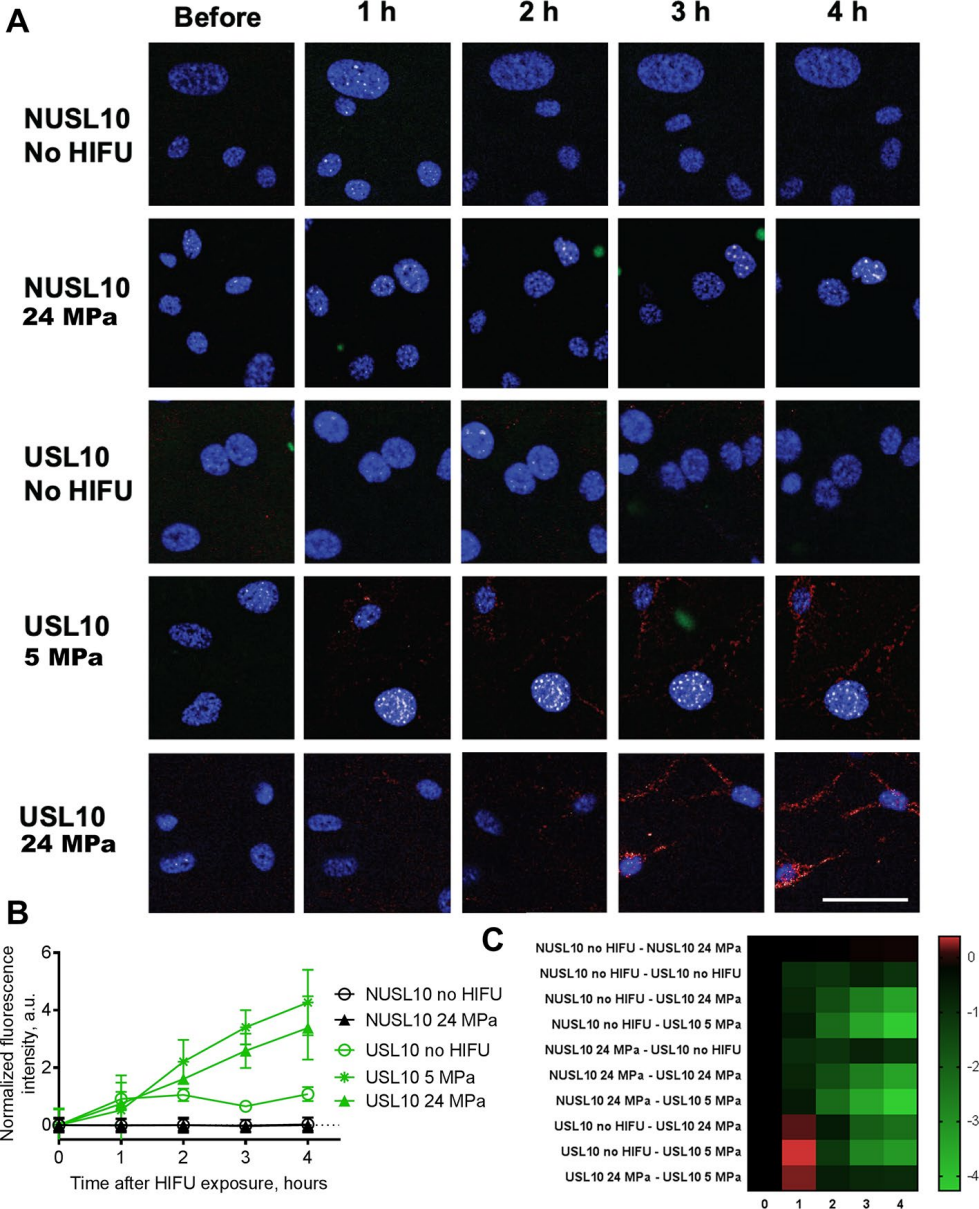
Uptake of ML1 released from NUSL10 and USL10 after HIFU treatment. Liposomal formulations were diluted in cell culture media and transferred without further processing onto CT26 and cells were evaluated for 4-h uptake of ML1 by live cell imaging **(A)**. For the uptake studies, liposomes were labeled with DiO’ (green) while ML1 was labeled with AF647 (red). Nuclei of CT26 were stained with Hoechst 33342 (blue) prior to addition of the preconditioned culture media. Scale bar is 50 micron and is applicable to all images. Timepoint “Before“ correspond to the point immediately before adding the liposomes, thus cells negative control. Semi-quantitative analysis of co-localization of the cell cytoplasm and AF647ML1 released from NUSL10 and USL10 is shown in the graph. In the graph **(B)**, the black lines correspond to NUSL10 with no HIFU treatment (empty circle symbol) and after 24 MPa 1 min HIFU (full triangle symbol). The green lines correspond to USL10 with no treatment (empty circle symbol), USL10 after 5 MPa 1 min HIFU (full square symbol) and USL10 after 24 MPa 1 min HIFU (full triangle symbol). The results of NUSL10 exposed to 5 MPa 1 min were comparable to those of NUSL10 exposed to 24 MPa 1 min and therefore omitted for clarity. Data are the average ± standard deviation of triplicate samples. The result of a 2-way ANOVA with Tukey’s multiple comparison test is presented as a heat map of the 95% confident intervals of the mean difference for all groups (i.e. 10) at all exposure times (i.e. 0, 1, 2, 3, and 4 h) **(C)**. Green indicates a significant difference between 2 groups.

**Figure 8** shows the live-cell confocal fluorescence microscopy pictures and semi-quantitative analysis of the uptake study. We detected cell-associated AF647-ML1 signal when the protein had been released from liposomes, or (in control experiments) added free AF647-ML1 to the cells. Although ML1 was released from NUSL10 after HIFU treatment (see **Figure 6**), the fluorophore amount (in AF647-ML1) was probably too low to be detected by the live cell imager. USL10 showed some spontaneous release without HIFU treatment which was only detected by the image analysis software. After HIFU treatment and at both acoustic conditions, ML1 released from USL10 was internalized by CT26, resulting in a punctuated red pattern in the cells cytoplasm. In the latest timepoint, ML1 was also found co-localized with the cell nucleus.

#### Cytotoxic Activity of ML1

**Figure 9:**
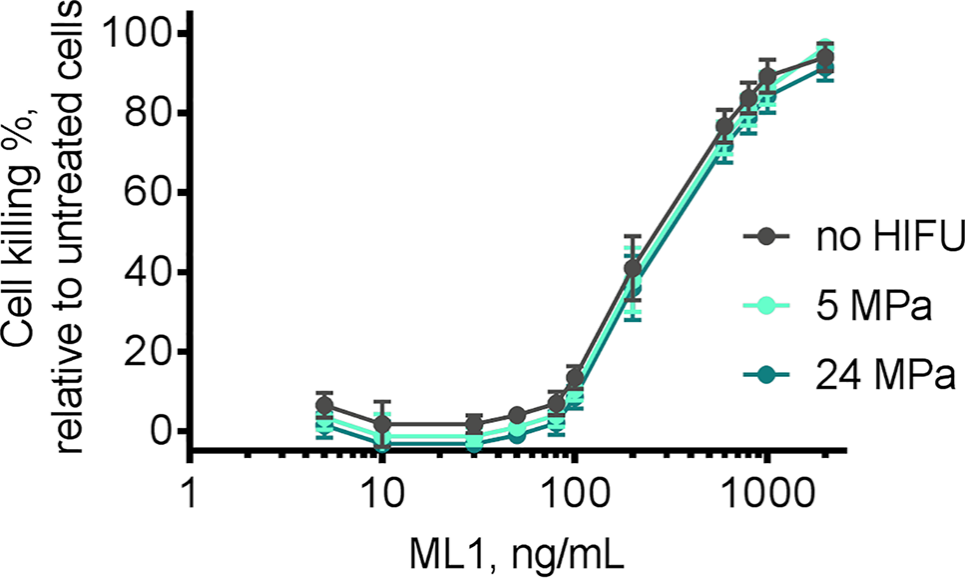
Cytotoxicity of free ML1 after exposure to HIFU 1 min negative pressures 5 MPa (light blue line) and 24 MPa (dark blue line). After HIFU treatment, ML1 was incubated with CT26 cells for 4 h, following which the medium was replaced by toxin-free medium. The IC_50_ was measured indirectly by MTS after 48 h. Free ML1 without any HIFU treatment was used as reference (grey line) and incubated with cells using the same protocol. Untreated cells were used as 0% killing control (n = 2). Untreated ML1 had an IC_50_ of 281 ng/ml, 5 MPa treated ML1 an IC_50_ of 316 ng/ml and 24 MPa ML1 an IC_50_ of 345 ng/ml.

CT26 cells are sensitive to ML1 in the low ng/ml range as shown before by us using similar assays (Beztsinna et al., 2018; Matos et al., 2018). Since we planned to refresh the media after 4 h of incubation with liposomes, we now evaluated cell death induced by ML1 after the 4-h exposure to liposomes followed by incubation with fresh culture medium for 44 h. We also investigated whether the treatment with HIFU would affect its cytotoxicity, in view of reports that ultrasound can lead to local heating (>40°C) (Ng and Liu, 2002), which may possibly inactivate ML1. We exposed free ML1 to two different peak negative pressures (5 and 24 MPa) for 1 min and tested different concentrations of the treated ML1 on CT26 cells. The cytotoxic profile is presented in **Figure 9** and resulted in quite similar dose-response curves and IC_50_ values of 280 to 350 ng/ml. We concluded that ML1 cytotoxicity is not influenced by the HIFU exposure at these experimental conditions, remaining constant around 300 ng/ml.

**Figure 10:**
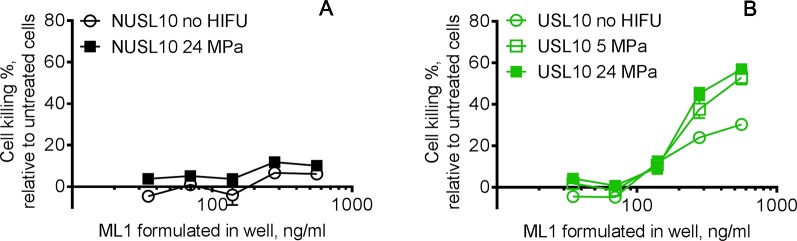
Bioactivity of released ML1 after USL10 and NUSL10 were exposed to HIFU. The released ML1 was in contact with cells for 4 h, then the medium was replaced by fresh medium and the cytotoxicity was measured 44 h later. **(A)** The black lines correspond to NUSL10 with no HIFU treatment (empty circle symbol) and after 24 MPa 1 min HIFU (full square symbol). **(B)** The green lines correspond to USL10 with no treatment (empty circle symbol), USL10 after 5 MPa 1 min HIFU (empty square symbol) and USL10 after 24 MPa 1 min HIFU (full square symbol). The cytotoxicity results of NUSL10 exposed to 5 MPa 1 min were comparable to those of NUSL10 exposed to 24 MPa 1 min and therefore omitted for clarity. For NUSL no significant differences were found between no HIFU and 24 MPa. For USL 5 and 24 MPa HIFU exposure leads to significant more cell killing compared to no HIFU at all ML1 concentrations, except for 139.6 ng/ml. Data are the average ± standard deviation of three independent experiments. A Wilcoxon matched-pairs signed rank test **(A)** and a 2-way ANOVA with Tukey’s multiple comparisons test **(B)** were performed.

As we demonstrated in the previous sections, only ML1-USL formulations were able to release ML1 when exposed to HIFU, while ML1-NUSL released the cytotoxic cargo to a much lower extent. This result was confirmed by the cytotoxicity evaluation of ML1 containing liposomes. Viability of CT26 after treatment with ML1-NUSL10 was only affected minimally, as only 10% cell killing was observed irrespective of HIFU had been applied (**Figure 10A**). These results are in good agreement with the live-cell imaging studies in which no uptake was visualized from ML1-NUSL10 (see **Figure 7**). Since ML1 is such a potent cytotoxin, the cell viability assay can detect the minor amount of release while the confocal fluorescence microscope was not sensitive enough to detect such low amounts of AF647ML1 (in the ng/ml range). When no HIFU was applied to ML1-USL10 (**Figure 10B**), there was 30% cell killing for the highest tested concentration. Extrapolation of the cell killing curve, indicates that it would require ca. 950 ng/ml of released ML1 to reach 50% cell killing. This is in line with the uptake quantification results (**Figure 8**) where the uptake difference was 4-fold different between the HIFU-exposed formulations and the non-treated formulation. Regarding ML1-USL10 after exposure to HIFU, potent cytotoxic activity was observed (**Figure 10B**), corresponding to IC_50_ values of 471 and 408 ng/ml for 5 MPa and for 24 MPa, respectively.

## Conclusion

We have demonstrated the potential of ultrasound sensitive liposomes as nanocarriers for high-molecular weight toxins like ML1. We tested three distinct formulations in terms of stability, release, and *in vitro* bioactivity. Overall, and as expected, the formulation containing the highest amount of PEG (i.e., USL20) is the most unstable in storage conditions and did not perform better than the two other counterparts with HIFU. The other two USLs, USL10 and USL5, complied with all requirements, i.e., a homogeneous size, stability, HIFU release, and *in vitro* tests. USL10 stood out as the one releasing higher amounts of ML1. Our experiments with CT26 cells confirmed that USL10-ML1 potently inhibited tumor cell viability after HIFU treatment. These promising results secure further investigation of these ultrasound sensitive formulations of ribosome-inactivating cytotoxins.

## Data Availability Statement

The datasets generated for this study are available on request to the corresponding author.

## Author Contributions

MM, RK, RD and GL designed the study and wrote the manuscript. MM performed all the formulation optimization studies, mistletoe lectin labeling, release, cell viability, and live-cell confocal imaging experiments, and analyzed the results. BM performed the transmission electron microscopy experiments. RD, BE and GL performed the acoustic experiment and results analysis. All authors discussed the results and commented on the manuscript.

## Conflict of Interest

The authors declare that the research was conducted in the absence of any commercial or financial relationships that could be construed as a potential conflict of interest.
